# Multi-Species Prediction of Physiological Traits with Hyperspectral Modeling

**DOI:** 10.3390/plants11050676

**Published:** 2022-03-01

**Authors:** Meng-Yang Lin, Valerie Lynch, Dongdong Ma, Hideki Maki, Jian Jin, Mitchell Tuinstra

**Affiliations:** 1 Department of Agronomy, Purdue University, West Lafayette, IN 47907, USA; lin805@purdue.edu (M.-Y.L.); vallynch24@gmail.com (V.L.); 2 Department of Agricultural and Biological Engineering, Purdue University, West Lafayette, IN 47907, USA; ma125@purdue.edu (D.M.); hmaki@purdue.edu (H.M.); jinjian@purdue.edu (J.J.)

**Keywords:** abiotic stress, corn, ecophysiology, high-throughput phenotyping, machine learning, nitrogen content, partial least square regression, relative water content, sorghum, remote sensing

## Abstract

Lack of high-throughput phenotyping is a bottleneck to breeding for abiotic stress tolerance in crop plants. Efficient and non-destructive hyperspectral imaging can quantify plant physiological traits under abiotic stresses; however, prediction models generally are developed for few genotypes of one species, limiting the broader applications of this technology. Therefore, the objective of this research was to explore the possibility of developing cross-species models to predict physiological traits (relative water content and nitrogen content) based on hyperspectral reflectance through partial least square regression for three genotypes of sorghum (*Sorghum bicolor* (L.) Moench) and six genotypes of corn (*Zea mays* L.) under varying water and nitrogen treatments. Multi-species models were predictive for the relative water content of sorghum and corn (R^2^ = 0.809), as well as for the nitrogen content of sorghum and corn (R^2^ = 0.637). Reflectances at 506, 535, 583, 627, 652, 694, 722, and 964 nm were responsive to changes in the relative water content, while the reflectances at 486, 521, 625, 680, 699, and 754 nm were responsive to changes in the nitrogen content. High-throughput hyperspectral imaging can be used to predict physiological status of plants across genotypes and some similar species with acceptable accuracy.

## 1. Introduction

Most agricultural production environments are exposed to abiotic stresses at one time or another, causing at least 50% economic loss in production each year [1]. Breeding for abiotic stress tolerant cultivars is essential for enhancing crop productivity and quality under global climate change [2]. However, breeding progress highly depends on the efficiency and accuracy of genotyping and phenotyping [3]. Genotyping is a high-throughput process that is well adapted to different species and accurate [4]. In contrast, improvements in phenotyping lag far behind. Traditional phenotyping methods are typically time consuming, labor intensive, species specific, destructive, and expensive. As a result, there is increasing interest in development of high-throughput phenotyping through machine learning-integrated remote sensing [5].

Water is required for normal plant growth and development. Plants have different strategies to adapt to water deficit, including changes in pigment composition and photosynthesis [6]. Water deficit decreases chlorophyll content in plants [7,8,9] due to degradation of light-harvesting chlorophyll a/b binding (LHCB) protein, the consequences of which are elevated chlorophyll a:b ratios [10]. Moreover, stomata close to reduce transpiration under water deficit, which limits carbon dioxide intake, slowing down carbon dioxide fixation. This leads to greater fluorescence, and heat is produced from chlorophyll absorbing similar amount of light [11]. Decreased carbon dioxide fixation further causes accumulation of electron donors in photosystem I, resulting in the over-production of reactive oxygen species [12], which induces the synthesis of antioxidants, such as carotenoid and anthocyanin [13].

Nitrogen is the most important nutrient for plants, accounting for 1.5% of dry matter [14]. Plants use an array of strategies to adapt for survival under nitrogen-limited condition. Since ammonia is required for biosynthesis of chlorophyll [15], plants inhibit chlorophyll accumulation under nitrogen deficiency [16], which indirectly reduces the abundance of the LHCB protein containing chlorophyll a and b [17]. Moreover, it is found that the expression of the LHCB protein is directly downregulated under nitrogen deficiency [18]. Further, plants accumulate anthocyanins to reduce oxidative damage [19] caused by production of reactive oxygen species under nitrogen deficiency [20].

Since plants produce specialized metabolites with unique reflectance characteristics under water or nitrogen stresses, reflectance with high-spectral resolution (hyperspectral reflectance, HR) is proposed as a method to predict water and nitrogen status of plants under water or nitrogen deficit. Predictive models have been developed for many plant species. The coefficient of determination (R^2^) is 0.98 for prediction of the relative water content (RWC) for five drought-sensitive and five drought-tolerant genotypes of rice [21]. R^2^ is 0.74 for the prediction of leaf water content (LWC) for four wheat genotypes [22]. The R^2^ is 0.81 and 0.92 for the prediction of LWC for two corn genotypes, separately [23]. Pre-dawn leaf water potential can be also predicted for three grapevine genotypes [24]. The fuel moisture content and equivalent water thickness of *Azalea japonica*, *Buxus sempervirens*, *Euonymus japonicus*, and *Ficus benjamina* can be also predicted, respectively [25]. On the other hand, canopy nitrogen content can be predicted for seven wheat genotypes [26]. Quantification of the nitrogen concentration in the leaf of one oilseed rape genotype [27] and estimation of the nitrogen concentration for one sugarcane genotype [28] can be also achieved by the predictive models.

However, models developed based on the responses of only one species and tested for prediction of the same species largely restrict the uses of these phenotyping models. In this study, HS imaging was used to study the water and nitrogen adaptation characteristics of corn and sorghum concerning the following questions: (1) the limitations of one genotype or species-based models, (2) the similarities in adaptation and genetics plays a role in prediction accuracy, (3) how cross-species models perform, and (4) the physiological basis of cross-species models. Three genotypes of sorghum and six genotypes of corn were selected for their adaptations to water and nitrogen deficits (Table 1). The relative water content (RWC) and nitrogen content (NC) were modeled with HR using partial least square regression (PLSR). Models were developed based on the responses of one genotype—combined sorghum, combined corn, or combined sorghum and corn—and used to predict RWC and NC. The R^2^ values are reported and compared for each model. Wavelengths important for RWC and NC were detected using coefficients and variable importance in projection (VIP) scores are reported.

## 2. Results

### 2.1. Diverse Responses of Sorghum and Corn to Water Deficit and Nitrogen Deficiency

Water and N treatment effects were significant in the study (Table 2). Regardless of genotypes, sorghum and corn had a significantly lower RWC under the water deficit condition compared to water sufficient condition. Similarly, sorghum and corn, regardless of genotypes, had a significantly lower NC under the nitrogen deficit condition compared to nitrogen sufficient condition.

The sorghum and corn genotypes were selected for this study based on differences in adaptation to drought and nitrogen stresses under field conditions (Table 1). Tx623 is susceptible to water deficit [29], while B35 and Tx7000 exhibit post-flowering tolerance [30] and pre-flowering tolerance [31] to water deficit, respectively. Moreover, B73xMo17, G80xPHP02, and BCC03xPHP02 are susceptible to water deficit [32], while PHJ33xPHP02 and CML550xPHP02 are tolerant to water deficit [33]. Although significant differences in RWC were not detected among sorghum or corn genotypes (Table 3), the observed plant responses should reflect that the range of physiological and spectral variation that are expected in these two important crop species.

Although there were few papers about nitrogen use efficiency of genotypes studied in this report (Table 1), lower NC in leaf tissue is associated with higher nitrogen use efficiency for both sorghum and corn. Tx7000 and CML550xPHP02 have high nitrogen use efficiency [34,35], while Tx623, B35, and B73xMo17 have low nitrogen use efficiency [36,37,38]. Tx7000 had lower NC in leaf tissue under nitrogen sufficient condition than Tx623 and B35, and CML550xPHP02 had lower NC in leaf tissue under nitrogen deficient condition than B73xMo17 (Table 3).

### 2.2. Prediction of Relative Water Content Using One Species Model

**Figure 1:**
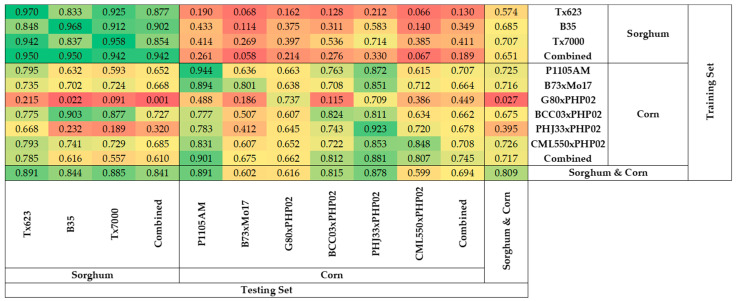
Heatmap of coefficients of determination of models predicting the relative water content of different genotypes with hyperspectral reflectance.

Sorghum models performed better in predicting the RWC of sorghum than in predicting the RWC of corn (Figure 1). The R^2^ of sorghum models to predict the RWC of sorghum ranged from 0.833 (Tx623 model to predict B35) to 0.970 (Tx623 model to predict Tx623), while the R^2^ for sorghum models to predict RWC of corn ranged from 0.066 (Tx623 model to predict CML550xPHP02) to 0.714 (Tx7000 model to predict PHJ33xPHP02). It was also clear that the combined sorghum model had a higher accuracy predicting the RWC of combined sorghum (R^2^ = 0.942) compared to predicting the RWC of combined corn (R^2^ = 0.189).

Similarly, corn models performed better in predicting the RWC of corn than in predicting the RWC of sorghum (Figure 1). The R^2^ for corn models to predict RWC of corn was between 0.115 (G80xPHP02 model to predict BCC03xPHP02) and 0.944 (P1105AM model to predict P1105AM), while the R^2^ for corn models to predict RWC of sorghum was between 0.022 (G80xPHP02 model to predict B35) and 0.903 (BCC03xPHP02 model to predict B35). The combined corn model had a higher accuracy predicting the RWC of combined corn (R^2^ = 0.745) compared to predicting the RWC of combined sorghum (R^2^ = 0.610).

### 2.3. Prediction of Relative Water Content Using a Two Species Model

**Figure 2:**
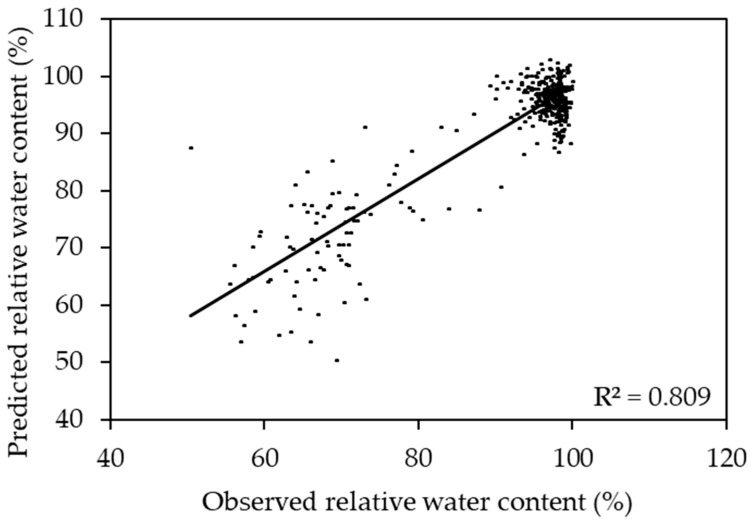
Scatter plot of observed vs. predicted relative water content using the sorghum- and corn-based model.

**Figure 3:**
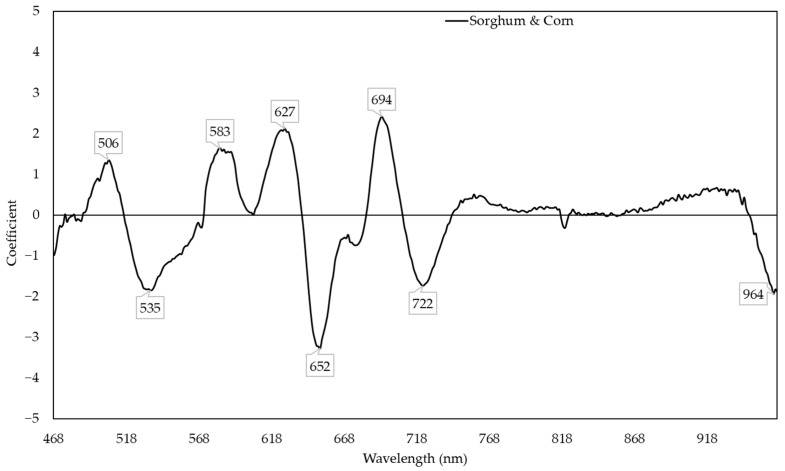
Coefficient in models predicting the relative water content with the responses of sorghum and corn.

**Figure 4:**
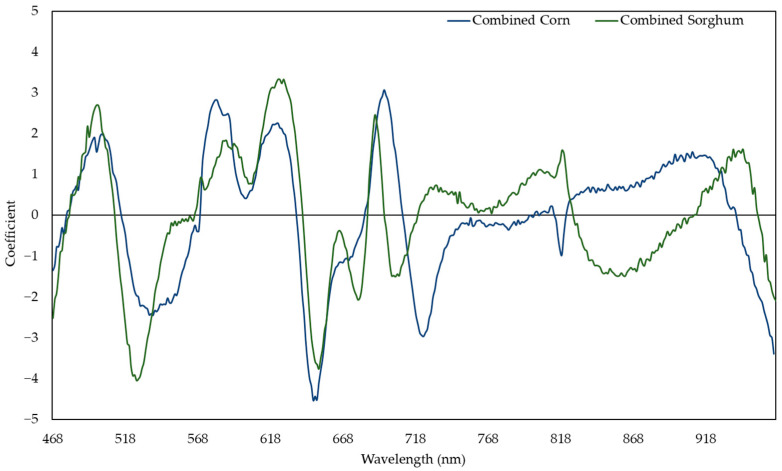
Coefficients in models predicting the relative water content with the responses of combined sorghum or combined corn.

The sorghum and corn model performed well in predicting both sorghum (R^2^ ranging from 0.844 to 0.891) and corn (R^2^ ranging from 0.599 to 0.891). Compared to other models, the sorghum and corn model had the highest R^2^ for prediction of the RWC of sorghum and corn (Figure 2). In this model, the reflectances around 506, 535, 583, 627, 652, 694, 722, and 964 nm were responsive to change in the RWC (Figure 3). The combined corn and combined sorghum models generally had a similar coefficient pattern in the visible light range (400–700 nm), but the coefficients in much of the near-infrared (NIR, 700–1000 nm) region had the opposite sign, resulting in a coefficient close to zero for sorghum and corn model (Figure 4).

### 2.4. Prediction of Nitrogen Content Using One Species Models

**Figure 5:**
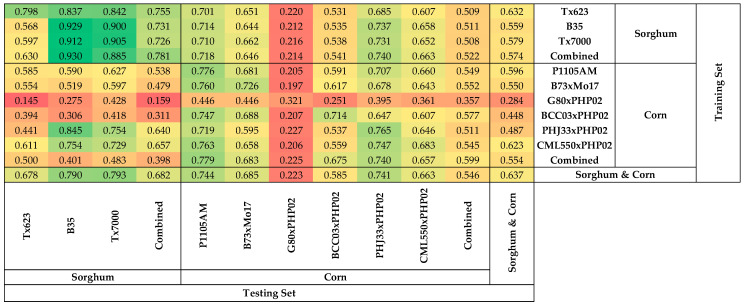
Heatmap of coefficients of determination of models predicting nitrogen content of different genotypes with hyperspectral reflectance.

Sorghum models performed better in predicting NC of sorghum than in predicting NC of corn (Figure 5). The R^2^ for the sorghum models to predict the nitrogen of sorghum was between 0.568 (B35 model to predict Tx623) and 0.929 (B35 model to predict B35), while the R^2^ for sorghum models to predict the NC of corn was between 0.212 (B35 model to predict G80xPHP02) and 0.737 (B35 model to predict PHJ33xPHP02). The combined sorghum model had a higher accuracy predicting the NC of combined sorghum (R^2^ = 0.781) compared to predicting the NC of combined corn (R^2^ = 0.522). Similarly, the combined corn model had a higher accuracy predicting the NC of combined corn (R^2^ = 0.599) compared to predicting the NC of combined sorghum (R^2^ = 0.398).

### 2.5. Prediction of Nitrogen Content Using Two Species Model

**Figure 6:**
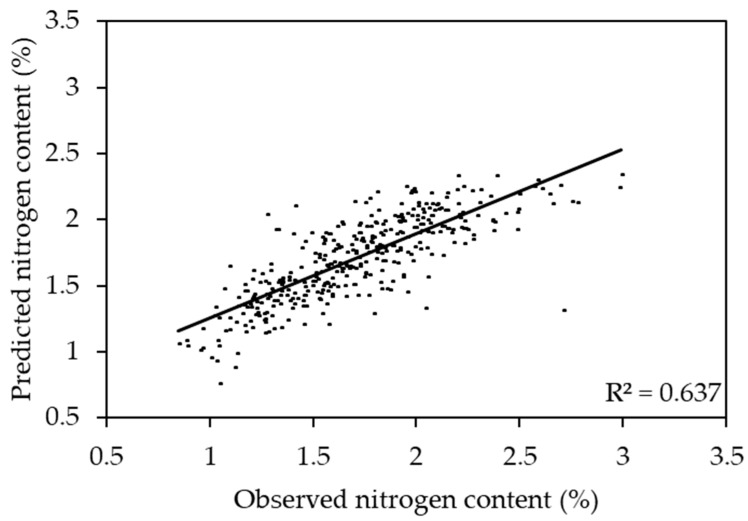
Scatter plot of observed vs. predicted nitrogen content using the sorghum- and corn-based model.

**Figure 7:**
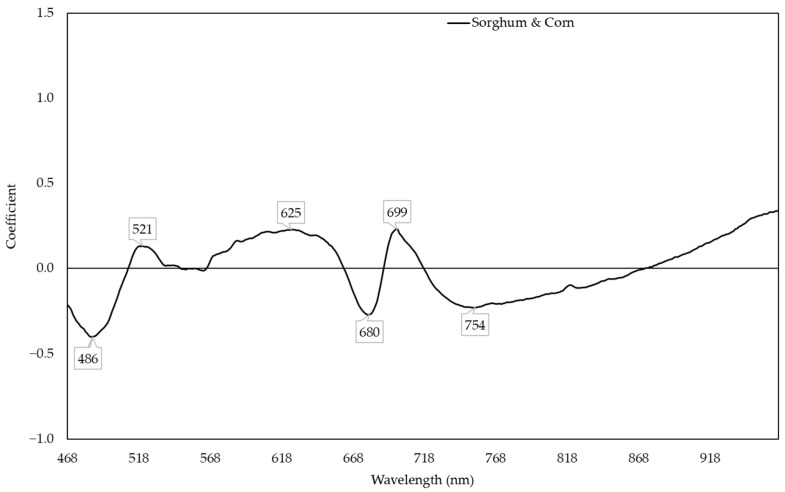
Coefficient in models predicting nitrogen content with the responses of sorghum and corn.

**Figure 8:**
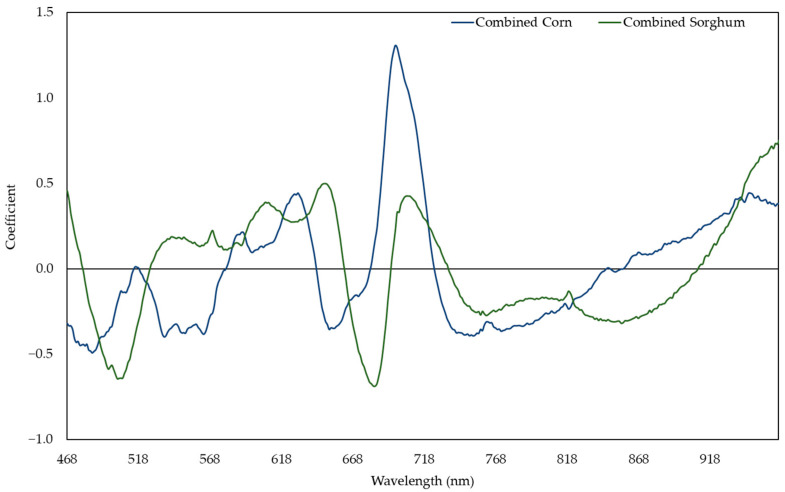
Coefficient in models predicting nitrogen content with the responses of combined sorghum or combined corn.

The sorghum and corn model performed well in predicting both sorghum (R^2^ ranging from 0.678 to 0.793) and corn (R^2^ ranging from 0.223 to 0.744) (Figure 5). The sorghum and corn model had the highest R^2^ to predict the NC of sorghum and corn (Figure 6). In this model, the reflectances around 486, 521, 625, 680, 699, and 754 nm were responsive to the change in NC (Figure 7). In general, the combined corn and combined sorghum models had a similar coefficient pattern to the sorghum and corn model, but the coefficients of the combined corn and combined sorghum models in part of the green region (500–600 nm) were significantly different (Figure 8).

## 3. Discussion

### 3.1. Sorghum and Corn Models for Predicting the Relative Water Content

The reflectances around 506, 535, 583, 627, 652, 694, 722, and 964 nm were responsive to changes in the RWC in the sorghum and corn model (Figure 3) and may be related to previously reported responses of pigments and photosynthetic activity to water deficit. R_506_ (R with subscript indicates reflectance at a certain wavelength) is associated with anthocyanin [39] and with water deficit inducing anthocyanin accumulation [13]. R_535_ and R_694_ are associated with chlorophyll and carotenoid [40,41] and with water deficit decreasing chlorophyll content while increasing the carotenoid content in corn [9,42], as well as with water deficit reducing chlorophyll content in sorghum [7,43]. R_627_ and R_652_ are associated with the chlorophyll b:a ratio [44], with the ratio decreasing in leaf tissues in response to water deficit in corn [45] and sorghum [46]. R_722_ is associated with the fluorescence of photosystem I [47], with chlorophyll a fluorescence increasing in response to water deficit due to a loss of photosystem I reaction centers [48]. R_964_ indicates a known water absorption peak [49].

The stepwise model (R^2^ = 0.810; Table S1) had a similar prediction accuracy compared to the PLSR model (R^2^ = 0.809; Figure 2), which can be a narrow-band spectral index for predicting the RWC. R_506_, R_583_, R_627_, and R_964_ were negatively correlated with RWC, while R_535_ and R_652_ were positively correlated with the RWC (Table S1). R_506_, R_583_, and R_627_ had positive coefficients while R_535_, R_652_, and R_964_ had negative coefficients in the PLSR model (Figure 3), which is consistent with R_506_, R_583_, and R_627_ having more negative slopes compared to R_535_, R_652_, and R_964_ in the stepwise model (Table S1). Moreover, R_694_ and R_722_ were dropped in the stepwise model because the coefficients of combined corn and combined sorghum models in the red edge range were incompatible (Figure 4). In fact, the coefficients of combined corn and combined sorghum models in most of NIR had opposite signs. Reflectance in NIR is related to leaf browning and necrosis [50] caused by phenolic compounds, such as *p*-coumaric acid and ferulic acid [51,52]. Additionally, reflectance in NIR is related to leaf structure [53,54], such as the ratio of mesophyll cell surface area exposed to intercellular air spaces per unit leaf surface area, leaf bi-coloration, and the presence of a thick leaf cuticle [55]. Further studies are needed to elucidate the differences in leaf browning, necrosis, and anatomy between corn and sorghum under water deficit. 

### 3.2. Sorghum and Corn Models for Predicting Nitrogen Content

Models for N in sorghum and corn showed that R_486_, R_521_, R_625_, R_680_, R_699_, and R_754_ were responsive to changes in the NC (Figure 7), which is consistent with previous reports [56,57]. R_699_ is associated with LHCB [58], which may be related to LHCB being downregulated by nitrogen deficiency in maize [18]. R_680_ and R_754_ are associated with chlorophyll content [59,60], which is consistent with chlorophyll content in the leaf decreasing under nitrogen deficiency in maize [61], as well as in sorghum [62].

The stepwise model (R^2^ = 0.634; Table S1) had similar prediction accuracy compared to the PLSR model (R^2^ = 0.637; Figure 6), which can be a narrow-band spectral index for predicting NC. R_521_ and R_625_ were negatively correlated with NC, while R_486_ and R_754_ were positively correlated with NC (Tables S1 and S2). R_521_ and R_625_ had positive coefficients, while R_486_ and R_754_ had negative coefficients in the PLSR model (Figure 7), which is consistent with R_521_ and R_625_ having more negative slopes compared to R_486_ and R_754_ in the stepwise model (Table S1). Moreover, R_680_ and R_699_ were dropped in stepwise model because the coefficients of combined corn and combined sorghum models in the red edge range were incompatible (Figure 8). The coefficients from 518 to 568 nm of the combined corn and combined sorghum models were significantly different (Figure 8), possibly due to different profiles of anthocyanin, which possesses an absorption maximum near 540–550 nm [63]. Sorghum produces an unique anthocyanin, 3-deoxyanthocyanidin, under nitrogen deficiency, which is not found in maize [64,65]. However, whether the existence of 3-deoxyanthocyanidin changes the coefficient pattern between 518 and 568 nm in the combined sorghum model needs to be further investigated.

### 3.3. Similarity in Adaptation and Genetics 

These studies showed that similarities in adaptation and genetic background do not necessarily ensure that models developed for one individual will perform well for other genotypes. B73xMo17, G80xPHP02, and BCC03xPHP02 are susceptible to water deficit (Table 1), but the R^2^ of the single genotype models ranged from 0.115 (G80xPHP02 model to predict BCC03xPHP02) to 0.824 (BCC03xPHP02 model to predict BCC03xPHP02) (Figure 1). These differences may indicate that the mechanisms for adaptation to water deficit vary among genotypes [66,67]. Genetic relatedness also does not ensure that models developed for one individual will perform well for other genotypes with a similar genetic background. In the PHP02 half-sibs, the R^2^ of the single genotype models varied significantly, ranging from 0.115 (G80xPHP02 model to predict BCC03xPHP02) to 0.923 (PHJ33xPHP02 model to predict PHJ33xPHP02) (Figure 1). This was surprising because each of these hybrids has one parent in common. This is similar to the finding that the transferability for predicting crop photosynthetic capacity does not necessarily depend on phylogenetic similarity [68].

### 3.4. Asymmetric Heatmap of Coefficients of Determination of the Models

Single genotype models may predict the responses of another genotype but not necessarily vice versa (Figure 1; Figure 5), and may depend on the intersection of wavelengths with higher VIP scores in the two models. For example, the Tx623 model predicted the RWC of B35 well (R^2^ = 0.833), and the B35 model predicted the RWC of Tx623 well (R^2^ = 0.848). This can be explained by the VIP scores over the wavelengths in the models predicting the RWC, where the responses of Tx623 and B35 were similar (Figure S1), suggesting that they have similar responses of reflectance to water deficit. On the other hand, the CML550xPHP02 model performed well predicting the RWC of Tx623 (R^2^ = 0.793), but the Tx623 model performed poorly predicting the RWC of CML550xPHP02 (R^2^ = 0.066). This is consistent with the observation that the CML550xPHP02 model contains more informative wavelengths with higher VIP scores compared to the Tx623 model (Figure S2), suggesting that CML550xPHP02 contains most of Tx623’s responsive reflectance to water deficit, but not vice versa.

### 3.5. Poor Performance in Predicting Nitrogen Content of G80xPHP02

The NC of G80xPHP02 was not predicted well using any model, and the G80xPHP02-based model cannot be used for predicting the NC of any genotypes, including itself (Figure 5). One of the reasons may be that reflectance changes are not correlated with the NC for G80xPHP02. The NC is the sum of all nitrogen compounds in plant tissue, so it is possible that G80xPHP02 has unique nitrogen-containing metabolites, whose reflectance cannot be captured by hyperspectral camera in visible-NIR range (350–1000 nm). Alternatively, the minor changes in metabolites only happen in a portion of the leaf, so the average reflectance of the whole shoot may not represent the phenotypic changes of G80xPHP02 in response to nitrogen deficiency. Follow-up studies on short-wave infrared range (1000–2500 nm) and more detailed image processing are needed. 

### 3.6. Non-Linear Models for Predicting the Relative Water Content and Nitrogen Content

The RWC and NC were also modeled using other machine learning algorithms including support vector machine regression (SVMR) and XGBoost regression (XGBR). SVMR exhibited the highest R^2^ cross-validated (CV) value, followed by XGBR and PLSR, which is consistent with PLSR having the highest root mean square error (RMSE), followed by XGBR and SVMR. On the other hand, SVMR had the highest absolute value of bias CV, followed by XGBR and PLSR. Both accuracy and bias should be considered for statistical model selection [69], which consequently depends on the application of the model. If the accuracy is important for application, the SVMR with the highest R^2^ CV and the lowest RMSE CV performs the best compared to XGBR and PLSR. However, if bias plays a role in application, the PLSR with the lowest absolute value of bias performs the best compared to XGBR and SVMR. Moreover, if biological meaning is essential for the application of the models, the PLSR is generally suggested, since the interpretability of non-linear models can be challenging [70].

## 4. Materials and Methods

### 4.1. Plant Material and Growing Condition

Three genotypes of sorghum (Tx623, B35, and Tx7000) and six genotypes of corn (P1105AM, B73xMo17, G80xPHP02, BCC03xPHP02, PHJ33xPHP02, and CML550xPHP02) were selected for their diverse adaptions to water and nitrogen stresses (Table 1). Phenotyping trials for sorghum were conducted in 2018 using a randomized complete block design with nine blocks, with each combination of three genotypes and four treatments represented in each block (Table S3). The corn phenotyping trails were conducted in 2017 using a randomized complete block design with 12 blocks, with each combination of six genotypes and four treatments represented in each block of the design (Table S3). In each trial, one plant was grown in a 5.7 L pot filled with the mixture of one-third topsoil, one-third sand, and one-third Turface Athletics™ MVP^®^ (Profile Products LLC, Buffalo Grove, IL, USA) with low water holding capacity and nutrient content but high cation exchange capacity. To develop models for plants with various water and nitrogen availability, treatments were factorial, including two levels of water conditions (well-watered or drought) and two levels of nitrogen conditions (nitrogen sufficient or deficient) (Table S3). The photoperiod was regulated using overhead high-pressure sodium lights from 6 am to 9 pm. Day and night temperatures were set at 24–32 °C and 24–27 °C, respectively.

From vegetative growth stage 4 until vegetative growth stage 6, all plants received 600 mL of water each day from the automated conveyor belt system. After vegetative growth stage 6, the plants assigned to receive well-watered and drought treatment were watered with 600 mL and 150 mL of water each day for a week, respectively. A volume of 12 mL of 1 M potassium phosphate monobasic solution, 6 mL of 0.33 M magnesium sulfate anhydrous solution, 8 mL of 0.017 M ferrous sulfate heptahydrate solution, 5 mL of 1 M calcium nitrate solution, and 2 mL of micronutrient stock solution (containing 50 mM of potassium chloride, 25 mM of boric acid, 2 mM of manganese sulfate tetrahydrate, 2 mM of zinc sulfate, 0.5 mM of cupric sulfate, and 0.5 mM of molybdic acid) was added to 1 L of deionized water as basic Hoagland’s solution. Volumes of 5 and 40 mL of 1 M ammonium nitrate solution were added into 1 L of basic Hoagland’s solution as the final solutions for the nitrogen deficient and sufficient treatments, respectively. Then, 300 mL of the final solutions were applied to each plant once a week over the course of the experiment. Phenotyping was conducted when plants reached V9.

### 4.2. Phenotyping Facility

An automated, high-throughput imaging system installed in a greenhouse at Purdue University was used in these studies [71]. The automated system consisted of a conveyor belt system that accommodated up to 108 pots in carriers with radio-frequency identification tags for high-throughput imaging. The pots were periodically relocated in the greenhouse by the conveyer to avoid ununiform plant growth caused by microclimates in the room [72]. The system included a hyperspectral imaging tower that accommodated imaging plants up to 1.5 meters tall from the top view and side view with MSV 500 hyperspectral cameras (Middleton Spectral Vision, Middleton, WI, USA) using push broom style scanning. The camera scanned from 400 to 1000 nm with an optical resolution down to 1.2 nm. Inside the imaging tower, there were eight studio halogen lamps to provide light for imaging. The towers had doors that automatically opened for moving plants and closed for phenotyping the plants, which took approximately 1 min per plant. The plants were automatically rotated on the imaging platform, so the widest plane of the plant was facing the camera. The top-view hyperspectral images were segmented into a black and white image to distinguish plant material from surrounding material based on the red edge (680–730 nm), which allowed for the calculation of the average reflectance of the top-view plant over wavelengths.

### 4.3. Ground Reference Data Collection

Further, 2.5 cm × 5.0 cm of top collar leaf was harvested for RWC measurement following the protocol below [73]. The fresh weight (FW) was determined immediately after harvesting. Then, the samples were submerged in deionized water overnight to obtain the turgid weight (TW). Finally, the dry weight (DW) was measured after drying in a 60 °C dryer overnight. All weights were measured using the AJ100L analytical balance (Mettler Toledo, Columbus, OH, USA) with a readability of 0.1 mg. RWC was determined using the following equation: RWC (%) = [(FW − DW)/(TW − DW)] ∗ 100%.

The NC of leaf samples was analyzed by a Thermo Scientific FlashEA 1112 Nitrogen and Carbon Analyzer for Soils, Sediments, and Filters (CE Elantech, Lakewood, NJ, USA) based on the flash dynamic combustion method described below. First, the gas mixture entered reactor one, a quart oxidation of the component, at 950 °C. Next, nitrogen oxides were reduced to elemental nitrogen in the copper-made reactor two at 840 °C. Finally, the sample was filtered through an adsorption filter, and the percentage of nitrogen in dry matter was determined by a gas chromatography column with thermal conductivity detector.

### 4.4. Statistical Analysis

The explanatory variables were reflectance from 468 to 966 nm with an interval of 1.34 nm, which were preprocessed through log (1/X) conversion and multiplicative signal correction with the mean and mean center. The response variables were RWC or NC, which were preprocessed through autoscaling. Partial least square regression (PLSR), support vector machine regression (SVMR), and XGBoost regression (XGBR) models were built and cross-validated (CV) with the leave-one-out method using PLS Toolbox 8.2.1 [74,75] in MATLAB R2018b (MathWorks, Natick, MA, USA). The number of latent variables used in models for the RWC and NC were six and three, respectively. The R^2^ and RMSE of models were calculated using the predicted and observed values (Equations (1) and (2)). The VIP scores and coefficients in models were also reported, and optimal wavelengths were selected based on the coefficient [21]. For further biological interpretation, stepwise regression models with optimal wavelengths were built using the mixed direction selection method with 0.25 as the *p*-value threshold for both entry and removal of dependent variables in JMP Pro 15 (SAS Institute, Cary, NC, USA).

(1) $$R^{2}=1-\frac{\sum {\left(y_{i}-{\hat{y}}_{i}\right)}^{2}}{\sum {\left(y_{i}-\bar{y}\right)}^{2}}$$

(2) $$\mathrm{RMSE}=\sqrt{\frac{1}{N}\times \sum {\left(y_{i}-{\hat{y}}_{i}\right)}^{2}}$$

## 5. Conclusions

Hyperspectral imaging is a non-destructive, high-throughput, and inexpensive method compared to traditional methods of quantifying plant water and nutrient status. This study demonstrated that multi-species models could be developed to predict water and nitrogen status of plants within and across these crop species with acceptable accuracy for agricultural uses. These models exhibit a broader application compared to previous studies using only few genotypes in one species.

## Figures and Tables

**Table 1:** Adaptation to water deficit and nitrogen use efficiency of the genotypes investigated in the study.

Plant Species	Genotype	Adaption to Water Deficit	Nitrogen Use Efficiency
Sorghum	Tx623	Susceptible	Low
	B35	Post-flowering tolerance	Low
	Tx7000	Pre-flowering tolerance	High
Corn	P1105AM	Unknown	Unknown
	B73xMo17	Susceptible	Low
	G80xPHP02	Susceptible	Unknown
	BCC03xPHP02	Susceptible	Unknown
	PHJ33xPHP02	Tolerant	Unknown
	CML550xPHP02	Tolerant	High

**Table 2:** Effects of treatments on relative water content and nitrogen content for sorghum and corn.

	Relative Water Content (%)			Nitrogen Content (%)		
Treatment	Water Sufficient	Water Deficient	Significance	Nitrogen Sufficient	Nitrogen Deficient	Significance
Sorghum	98.4 ± 0.54 a	66.0 ± 5.29 b	***	1.81 ± 0.18 a	1.16 ± 0.13 b	***
Corn	97.0 ± 1.60 a	91.0 ± 11.10 b	***	1.99 ± 0.34 a	1.59 ± 0.28 b	***

Each value in the table is the mean ± standard deviation for each treatment. One-way analysis of variance (ANOVA) was used to determine significant differences among the treatments. * 0.01< *p* ≤ 0.05; ** 0.001 < *p* ≤ 0.01; *** *p* ≤ 0.001; NS, nonsignificant at *p* > 0.05. Values with different letters within the row are significantly different among the water or nitrogen treatments determined by Student’s *t* test (α = 0.05).

**Table 3:** Effects of genotypes on relative water content and nitrogen content under different water and nitrogen conditions.

		Relative Water Content (%)		Nitrogen Content (%)	
Species	Genotypes	Water Sufficient	Water Deficient	Nitrogen Sufficient	Nitrogen Deficient
Sorghum	Tx623	98.4 ± 0.51	67.2 ± 5.43	1.82 ± 0.08 a	1.19 ± 0.10
	B35	98.5 ± 0.59	66.1 ± 5.03	1.92 ± 0.22 a	1.15 ± 0.13
	Tx7000	98.5 ± 0.54	64.7 ± 5.37	1.69 ± 0.15 b	1.13 ± 0.15
	**Significance**	NS	NS	***	NS
Corn	P1105AM	97.7 ± 1.05 a	90.6 ± 11.6	2.16 ± 0.35	1.70 ± 0.27 a
	B73xMo17	95.4 ± 1.42 c	92.1 ± 6.2	2.00 ± 0.35	1.67 ± 0.29 a
	G80xPHP02	97.6 ± 1.27 ab	89.0 ± 13.7	1.91 ± 0.28	1.65 ± 0.36 a
	BCC03xPHP02	96.8 ± 1.81 b	90.2 ± 12.4	1.96 ± 0.28	1.57 ± 0.20 ab
	PHJ33xPHP02	97.5 ± 1.13 ab	89.3 ± 13.6	2.02 ± 0.36	1.46 ± 0.19 b
	CML550xPHP02	97.2 ± 1.61 ab	95.2 ± 6.2	1.89 ± 0.35	1.47 ± 0.25 b
	**Significance**	***	NS	NS	*

Each value in the table is the mean ± standard deviation for each treatment. One-way analysis of variance (ANOVA) was used to determine significant differences among the treatments. * 0.01< *p* ≤ 0.05; ** 0.001 < *p* ≤ 0.01; *** *p* ≤ 0.001; NS, nonsignificant at *p* > 0.05. Values with different letters within the row are significantly different among the water or nitrogen treatments determined by Student’s *t* test (α = 0.05).

## Data Availability

Data and meta-data are available at The Purdue University Research Repository (PURR), https://purr.purdue.edu/publications/3958/1 (accessed on 30 January 2022).

## Supplementary Materials

The following are available online at https://www.mdpi.com/article/10.3390/plants11050676/s1, Table S1: Stepwise regression for reflectance at optimal wavelength and trait, Table S2: Evaluation of models built with different machine learning algorithms, Table S3. Number of samples in each treatment, Figure S1: Variable importance in projection (VIP) scores in models predicting the relative water content with the responses of Tx623 and B35, Figure S2: Variable importance in projection (VIP) scores in models predicting the relative water content with the responses of Tx623 and CML550xPHP02.
