# Supplementary material for: Multi-Species Prediction of Physiological Traits with Hyperspectral Modeling
Source: Plants (Basel). 2022 Mar 1;11(5):676. doi: 10.3390/plants11050676 (PMC8912614; doi:10.3390/plants11050676)
Supplement: Supplementary file 1 [file plants-11-00676-s001.zip › plants-1601172-supplementary.pdf]

plants

Supplementary Materials

Table S1. Stepwise regression for reflectance at optimal wavelength and trait.

Traits	Dependent Variables	Significance	Slope
**Relative water content**	R_{506}	***	−6.53
	R_{535}	***	+7.28
	R_{583}	*	−2.95
	R_{627}	**	−10.4
	R_{652}	***	+11.0
	R_{694}	NS	0
	R_{722}	NS	0
	R_{964}	**	−0.277
R^2	0.810		
**Nitrogen content**	R_{486}	***	+0.168
	R_{521}	**	−0.0521
	R_{625}	***	−0.0936
	R_{680}	NS	0
	R_{699}	NS	0
	R_{754}	***	+0.0132
R^2	0.634		

R^2, coefficient of determination; R with number as subscript, reflectance at certain wavelength; F-test was used to determine linear relationship between reflectance at selected wavelength and trait. * $0.01 < P \le 0.25$; ** $0.001 < P \le 0.01$; *** $P \le 0.001$; NS, nonsignificant at $P > 0.25$. Slope in the linear equation was also reported.

Table S2. Evaluation of models built with different machine learning algorisms.

Traits	Algorisms	R^2 C	R^2 CV	RMSEC	RMSECV	Bias C	Bias CV
**RWC**	PLSR	0.809	0.793	5.48	5.70	0	−0.00326
	XGBR	0.995	0.799	0.874	5.65	0.00109	−0.0492
	SVMR	0.908	0.874	3.83	4.46	0.329	0.325
**NC**	PLSR	0.637	0.626	0.238	0.241	1.78×10^{-15}	−0.000152
	XGBR	0.968	0.702	0.0742	0.215	0.000171	0.00252
	SVMR	0.849	0.772	0.153	0.189	−0.00368	−0.00341

RMSE, root-mean-square error; R^2, coefficient of determination; C, calibration; CV, cross validation; RWC, relative water content; NC, nitrogen content; PLSR, partial least squares regression; SVMR, support vector machine regression; XGBR, XGBoost regression.

Table S3. Number of samples in each treatment.

Plant Species	Genotype	Water Sufficient Nitrogen Sufficient	Water Sufficient Nitrogen Deficient	Water deficient Nitrogen Sufficient	Water Deficient Nitrogen Deficient
Sorghum	Tx623	9	9	9	9
	B35	9	9	8	9
	Tx7000	9	9	9	9
Corn	P1105AM	12	10	12	11
	B73xMo17	12	12	12	12
	G80xPHP02	11	12	12	11
	BCC03xPHP02	11	11	11	11
	PHJ33xPHP02	11	12	12	11
	CML550xPHP02	12	10	11	8

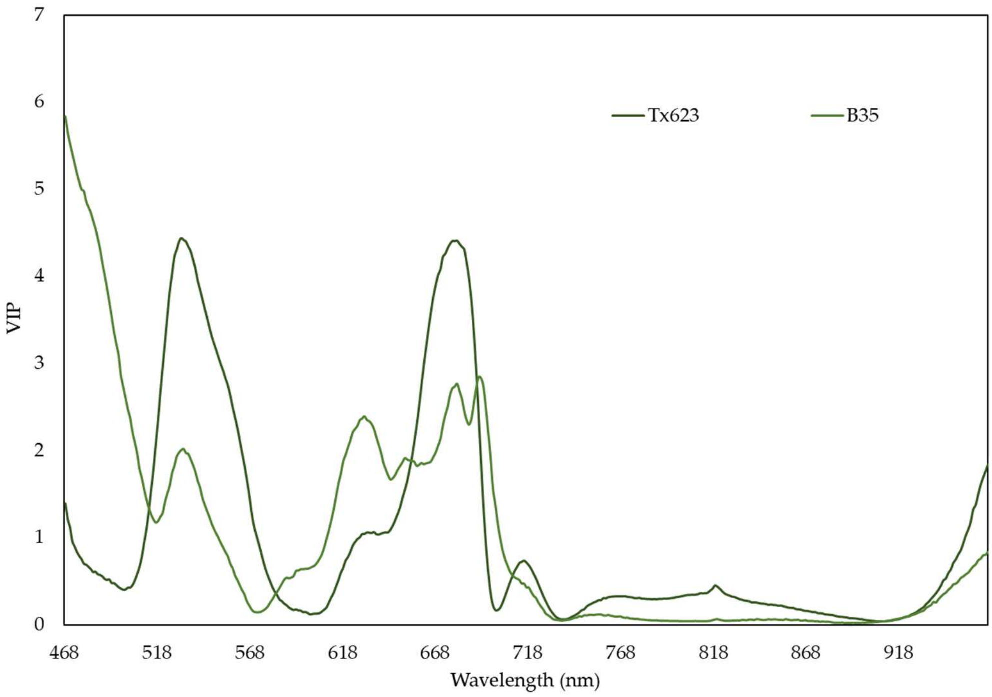

Figure S1. Variable importance in projection (VIP) scores in models predicting relative water content with the responses of Tx623 and B35.

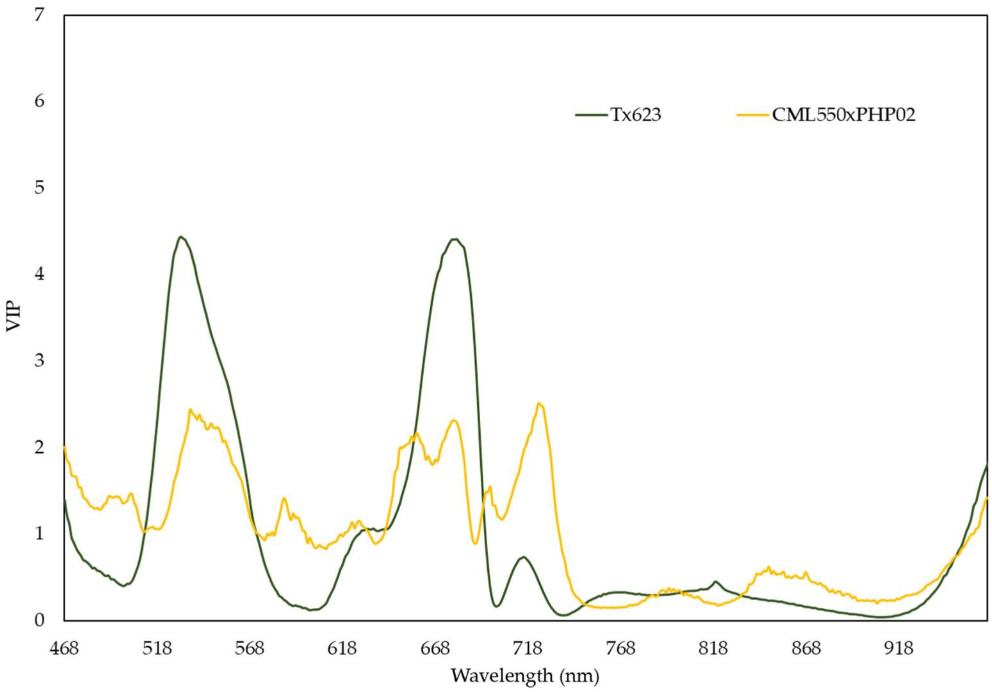

Figure S2. Variable importance in projection (VIP) scores in models predicting relative water content with the responses of Tx623 and CML550xPHP02.
